# Triglyceride Threshold for Intensive Lipid-Lowering Therapy in Acute Hyper-Triglyceridemic Pancreatitis: A Retrospective Study

**DOI:** 10.3390/jcm15124581

**Published:** 2026-06-12

**Authors:** Meiling Yu, Enqiang Mao, Yanyan Xu, Tongtian Ni

**Affiliations:** 1 Department of Emergency, Ruijin Hospital, Shanghai Jiao Tong University School of Medicine, Shanghai 200025, China; yml11851@rjh.com.cn (M.Y.); maoeq@yeah.net (E.M.); 2 College of Health Sciences and Technology, Shanghai jiao Tong University School of Medicine, Shanghai 200025, China; 3 Department of Laboratory Medicine, Ruijin Hospital, Shanghai Jiao Tong University School of Medicine, Shanghai 200025, China

**Keywords:** hyper-triglyceridemic acute pancreatitis, triglyceride reduction, disease severity, nonlinear relationship, prognosis

## Abstract

**Background**: The prognostic significance of early triglyceride (TG) reduction in hyper-triglyceridemic acute pancreatitis (HTG-AP) remains controversial. This study aimed to investigate the association between admission TG levels, early TG goal achievement, and clinical outcomes in patients with HTG-AP. **Methods**: This retrospective study included 345 patients with HTG-AP. Early TG goal achievement was defined as TG ≤ 5.65 mmol/L within 72 h of admission. Disease severity was classified according to the Revised Atlanta Classification. Associations between TG parameters and outcomes were analyzed using ordinal logistic regression. **Results**: No significant differences were observed between the goal-achieved (*n* = 274) and non-goal-achieved (*n* = 71) groups regarding pancreatic necrosis, hospital stay, or in-hospital mortality. Admission TG levels demonstrated that the highest TG levels did not correspond to the most severe disease course. Compared to patients with admission TG > 90.4 mmol/L, those with TG levels between 11.3 and 56.5 mmol/L had significantly lower odds of more severe disease, while no significant differences were found for TG levels between 56.5 and 90.4 mmol/L. Neither admission TG levels nor the degree of TG reduction within 72 h was significantly correlated with pancreatic necrosis. **Conclusions**: Early TG goal achievement was not associated with improved clinical outcomes in patients with HTG-AP. The routine use of aggressive early lipid-lowering therapy requires further validation before it can be routinely recommended. TG ≥ 56.5 mmol/L might represent a threshold for disease progression risk. Further studies are needed to determine the clinically meaningful cutoff value.

## 1. Introduction

Hypertriglyceridemia has emerged as the third-most common etiology of acute pancreatitis worldwide, following gallstones and alcohol consumption, and is increasingly recognized as a leading cause of recurrent pancreatitis in specialized lipid clinics [1]. The global pooled prevalence of hyper-triglyceridemic acute pancreatitis (HTG-AP) is estimated at 11.6%, with marked geographical variation: Eastern countries report a significantly higher proportion of 16.3% compared to 5.4% in Western countries [2].

With changing lifestyles and dietary patterns, the prevalence of HTG-AP in China has demonstrated a significant increasing trend over the past two decades [3]. In some Chinese centers, HTG-AP accounts for up to 60% of acute pancreatitis cases, making it the second leading etiology after biliary pancreatitis [4]. Compared to acute pancreatitis due to other etiologies, HTG-AP patients experience higher mortality rates, increased disease severity, and significantly higher recurrence rates [3].

The mechanistic link between serum triglyceride (TG) levels and pancreatitis severity has been well established. Elevated triglyceride levels are positively associated with the risk of acute pancreatitis [5]. Elevated serum triglyceride concentrations are associated with an increased risk of complications including pancreatic necrosis, systemic inflammatory response syndrome, shock, and multi-organ failure [6]. The underlying pathophysiology is primarily attributed to the hydrolysis of triglycerides by pancreatic lipase, generating high concentrations of free fatty acids that exceed albumin-binding capacity, leading to microcirculatory damage, calcium overload, and activation of inflammatory cascades [7,8,9].

Given the association between triglyceride levels and disease severity, rapid triglyceride reduction has been hypothesized as a therapeutic strategy to improve clinical outcomes. Interventions including insulin, heparin, and plasmapheresis are commonly used to achieve rapid triglyceride lowering [10,11,12,13]. However, although these interventions effectively reduce triglyceride concentrations, they generally do not reduce acute pancreatitis-related complications or accelerate disease recovery [14]. Therefore, the role of aggressive lipid-lowering therapy in the acute setting remains controversial.

Despite these observations, several important questions remain unanswered. While previous studies have compared specific lipid-lowering modalities, it remains unclear whether achieving a predefined TG target within 72 h, regardless of the method used, is associated with improved clinical outcomes. Additionally, the quantitative relationship between admission TG levels, particularly across different strata of extreme hypertriglyceridemia, and disease severity has not been well characterized. These knowledge gaps have led to considerable practice variation in the clinical management of HTG-AP, highlighting the need for real-world evidence to inform clinical decision-making.

This study aims to investigate the association between admission triglyceride levels, early triglyceride goal achievement, and clinical outcomes in patients with HTG-AP, as well as the relationship between triglyceride parameters and disease severity, to inform clinical risk stratification and therapeutic decision-making.

## 2. Methods

### 2.1. Study Subjects

This single-center retrospective study from Ruijin Hospital enrolled patients from 1 January 2013 to 31 December 2025. The study was approved by the Ethics Committee of Ruijin Hospital affiliated with Shanghai Jiao Tong University School of Medicine (2026256) and written informed consent was waived. The whole research process and data analysis were performed in accordance with the principles expressed in the 1964 Helsinki Declaration and its later amendments.

### 2.2. Study Population

Inclusion criteria: (1) Patients with a diagnosis of HTG-AP according to the Revised Atlanta Classification criteria [15]; (2) HTG-AP was defined as acute pancreatitis with serum triglyceride levels ≥ 11.3 mmol/L on admission [16], in the absence of other identifiable etiologies (gallstones, alcohol, drugs, or anatomical abnormalities, etc.).

Exclusion criteria: (1) age < 18 years; (2) pregnancy; (3) recurrent pancreatitis; (4) severe heart, liver, or kidney dysfunction; (5) malignant tumor; (6) triglyceride measurement > 24 h after disease onset; and (7) missing critical data.

### 2.3. Data Collection

Demographic and clinical data were extracted from electronic medical records. Collected variables included: age, sex, body mass index (BMI), medical history (hypertension, diabetes mellitus, drinking, current smoke), laboratory parameters on admission (white blood cell count, C-reactive protein, procalcitonin, alanine aminotransferase, total bilirubin, albumin, blood urea nitrogen, serum creatinine, blood glucose, lactic acid, and triglyceride levels), and triglyceride levels at 72 h after admission. The triglyceride-lowering intervention measures included: fenofibrate administration, heparin/low-molecular-weight heparin administration, insulin therapy, and double-filtration plasmapheresis (DFPP). Patients received triglyceride-lowering interventions according to the discretion of the attending physician.

### 2.4. Group Definitions

Triglyceride goal achievement: Patients were dichotomized into the goal-achieved group (triglyceride ≤ 5.65 mmol/L at 72 h after admission) and the non-goal-achieved group (triglyceride > 5.65 mmol/L at 72 h after admission). The threshold of 5.65 mmol/L was chosen based on previous studies identifying this level as a risk threshold for acute pancreatitis and its recurrence [17,18], as well as relevant guideline recommendations [19].

Triglyceride parameters: The following parameters were calculated for each patient: (1) ΔTG = TG on admission − TG at 72 h after admission and (2) percentage decrease = (ΔTG/TG on admission) × 100%.

Severity Classification: Disease severity was classified according to the Revised Atlanta Classification into three categories: mild acute pancreatitis (MAP, absence of organ failure and local or systemic complications), moderately severe acute pancreatitis (MSAP, transient organ failure lasting < 48 h or local/systemic complications), and severe acute pancreatitis (SAP, persistent organ failure lasting > 48 h). Organ failure was defined as a modified Marshall score of ≥ 2 for the respiratory, cardiovascular, or renal system [15].

Pancreatic necrosis was assessed by contrast-enhanced computed tomography and categorized as none, <30%, 30–50%, or >50% of pancreatic parenchymal involvement [20].

### 2.5. Statistical Analysis

Continuous variables were expressed as mean ± standard deviation (SD) for normally distributed data or as median with interquartile range (IQR) for non-normally distributed data. Categorical variables were expressed as frequencies and percentages. Comparisons between groups were performed using Student’s *t*-test or the Mann–Whitney U test for continuous variables and the chi-square test or Fisher’s exact test for categorical variables, as appropriate. For comparisons among three severity groups, the Kruskal–Wallis test was used.

Ordinal logistic regression was used to analyze the association between admission triglyceride levels and disease severity. Ordinal logistic regression is based on the proportional odds assumption, meaning that the effect of each predictor on disease severity is consistent across different severity thresholds. For analysis of the association between admission triglyceride levels and disease severity, TG levels were categorized into eight strata: (1) the diagnostic threshold for HTG-AP (≥11.3 mmol/L) served as the lowest boundary; (2) intervals of approximately 11.3 mmol/L were used to create equally spaced strata to explore potential threshold effects while maintaining adequate sample sizes within each stratum; and (3) the highest stratum (TG > 90.4 mmol/L, corresponding to eight times the diagnostic threshold) was used as the reference group to assess risk at extreme TG levels. All baseline variables, including demographic characteristics, medical history, and admission laboratory parameters, were included as covariates using a full-variable inclusion strategy to avoid omission of potential confounders.

All statistical analyses were performed using SPSS 29.0 statistical software. *p* value < 0.05 was considered statistically significant. The STROBE Statement checklist is available in the Supplementary Materials.

## 3. Results

### 3.1. Clinical Characteristics of the Patients

**Figure 1:**
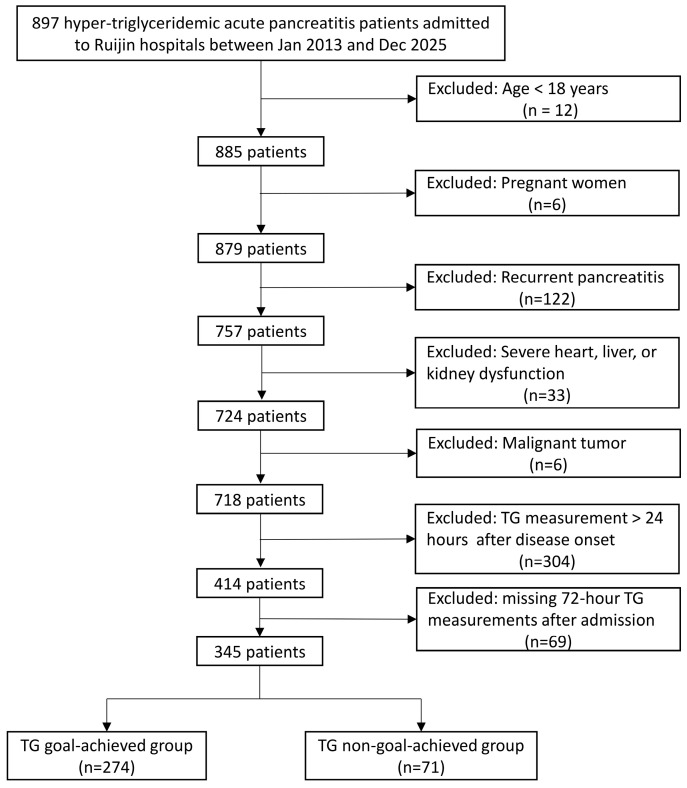
Flowchart of patient selection for hyper-triglyceridemic acute pancreatitis. Note: TG: Triglyceride.

Figure 1 shows the screening process for a total of 897 patients with hyper-triglyceridemic acute pancreatitis admitted to Ruijin Hospital between January 2013 and December 2025. Patients were sequentially excluded based on the following criteria: age < 18 years (*n* = 12), pregnancy (*n* = 6), recurrent pancreatitis (*n* = 122), severe heart, liver, or kidney dysfunction (*n* = 33), malignant tumor (*n* = 6), triglyceride measurement > 24 h after disease onset (*n* = 304), and missing 72 h TG measurements after admission (*n* = 69). After sequential exclusions, 345 patients were finally included and divided into two groups: the triglyceride goal-achieved group (*n* = 274) and the triglyceride non-goal-achieved group (*n* = 71).

Table 1 compares the baseline characteristics between the triglyceride goal-achieved group and the non-goal-achieved group. No statistically significant differences were observed between the two groups in terms of age, sex, body mass index, medical history, most laboratory parameters (white blood cell count, C-reactive protein, procalcitonin, albumin, blood urea nitrogen, serum creatinine, blood glucose, and lactic acid), or disease severity scores. Statistically significant differences were found in alanine aminotransferase (*p* = 0.031) and total bilirubin (*p* = 0.002) levels between the two groups. Regarding triglyceride-lowering interventions, no significant differences were observed in the proportions of patients receiving fenofibrate, heparin/low-molecular-weight heparin, or insulin between the two groups, whereas the proportion of patients receiving DFPP differed significantly (0.73% vs. 7.04%, *p* < 0.001).

### 3.2. The Relationship Between Early Triglyceride Goal Achievement and Clinical Outcomes

Table 2 shows that no statistically significant differences were observed between the triglyceride goal-achieved group and the non-goal-achieved group in terms of pancreatic necrosis, length of hospital stay, or in-hospital mortality. The overall in-hospital mortality rate was extremely low, with only 4 deaths occurring in the entire cohort.

### 3.3. The Relationship Between Admission Triglyceride Levels and Disease Severity

Table 3 demonstrates that statistically significant differences were observed among patients with different disease severity groups in triglyceride levels on admission, the difference in triglyceride levels between admission and 72 h after admission (ΔTG), and the percentage reduction in triglyceride levels. Among the three groups, the MSAP group had the highest triglyceride level on admission, followed by the SAP group, whereas the MAP group had the lowest. The trend of ΔTG was consistent with that of triglyceride levels on admission, with the MSAP group showing the largest reduction, followed by the SAP group, and the MAP group showing the smallest reduction. Regarding the percentage reduction in triglyceride levels, the MSAP group had the highest percentage, followed by the SAP group, whereas the MAP group had the lowest. However, no statistically significant difference was observed in triglyceride levels at 72 h after admission among the three groups.

**Figure 2:**
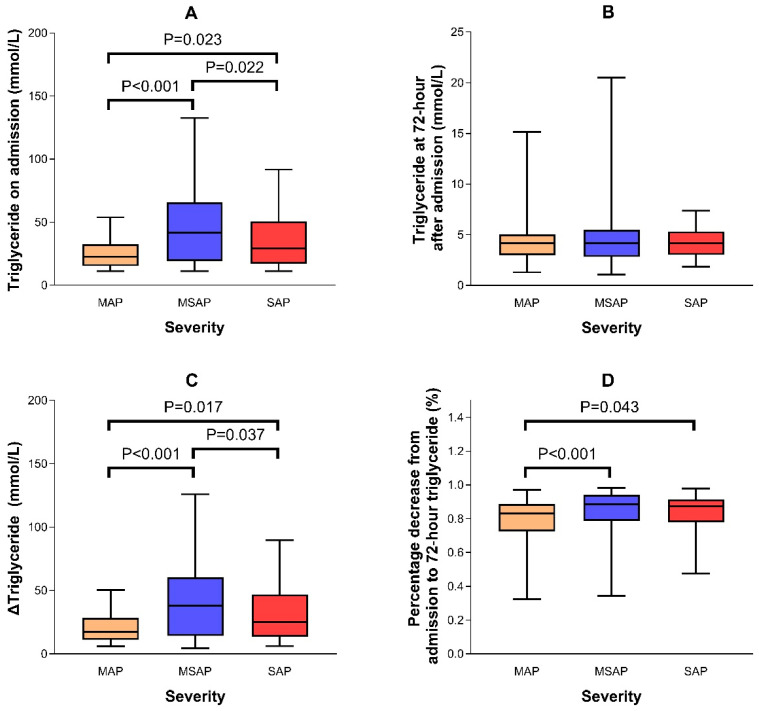
The relationship between disease severity and triglyceride parameters: triglyceride levels on admission (**A**), triglyceride levels at 72 h after admission (**B**), the difference between admission and 72 h triglyceride levels (**C**), and the percentage decrease from admission to 72 h (**D**).

Figure 2 illustrates the relationship between disease severity and triglyceride parameters. Figure 2A shows that, at admission, triglyceride levels in the MAP group were significantly lower than those in the MSAP and SAP groups (*p* < 0.05). Additionally, triglyceride levels in the MSAP group were significantly higher than those in the SAP group (*p* < 0.05). Figure 2B demonstrates that, after 72 h of treatment, no significant differences in triglyceride levels were observed among the MAP, MSAP, and SAP groups (*p* > 0.05). Figure 2C compares the difference in triglyceride levels between admission and 72 h after treatment among the three groups. The ΔTG in the MAP group was significantly lower than that in the MSAP and SAP groups (*p* < 0.05), while the ΔTG in the MSAP group was significantly higher than that in the SAP group (*p* < 0.05). Figure 2D presents the percentage decrease in triglyceride levels from admission to 72 h after treatment among the three groups. The percentage decrease in the MAP group was significantly lower than that in the MSAP and SAP groups (*p* < 0.05).

Table 4 presents the results of ordinal logistic regression with disease severity as the dependent variable. Using the TG > 90.4 mmol/L group as the reference, after adjusting for confounding factors, triglyceride levels on admission were significantly associated with disease severity. The results showed that compared with the TG > 90.4 mmol/L group, patients with triglyceride levels in the ranges of 11.3–22.6 mmol/L, 22.6–33.9 mmol/L, and 33.9–45.2 mmol/L had a lower risk of developing more severe pancreatitis. No statistically significant differences in disease severity were observed between the remaining triglyceride strata (45.2–56.5 mmol/L, 56.5–67.8 mmol/L, 67.8–79.1 mmol/L, and 79.1–90.4 mmol/L) and the reference group. All VIF values of confounding factors were less than 5, indicating no collinearity issues in the model (Table 5).

### 3.4. The Relationship Between Pancreatic Necrosis and Triglyceride Parameters

**Figure 3:**
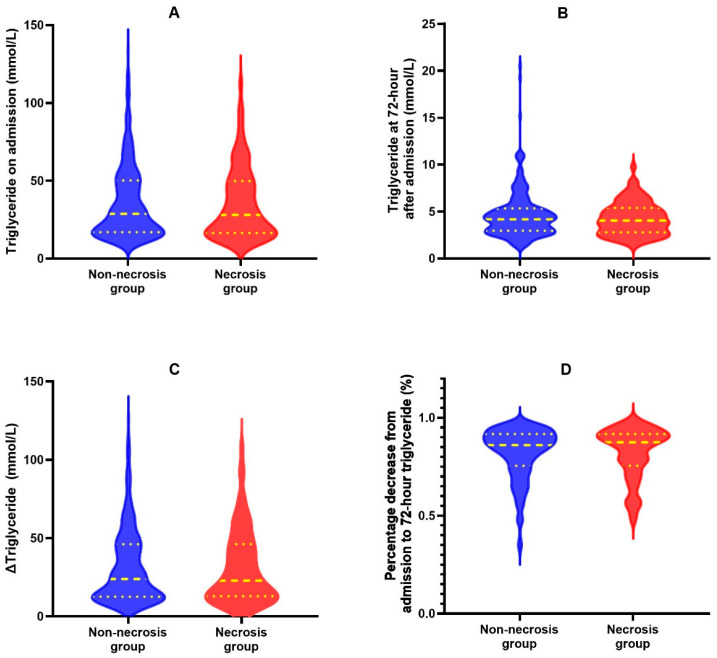
The relationship between pancreatic necrosis and triglyceride parameters: triglyceride levels on admission (**A**), triglyceride levels at 72 h after admission (**B**), the difference between admission and 72 h triglyceride levels (**C**), and the percentage decrease from admission to 72 h (**D**).

Figure 3 shows the comparison of triglyceride parameters between patients with and without pancreatic necrosis. Figure 3A illustrates triglyceride levels on admission, Figure 3B presents triglyceride levels at 72 h after admission, Figure 3C displays the absolute decrease in triglyceride levels, and Figure 3D depicts the percentage decrease in triglyceride levels. No statistically significant differences were observed between the two groups for any of the parameters.

## 4. Discussion

In this retrospective study, we observed that early failure to achieve the triglyceride goal (reduction to ≤5.65 mmol/L within 72 h) was not significantly associated with adverse clinical outcomes. Admission triglyceride levels demonstrated that the highest triglyceride levels did not correspond to the most severe disease course. Compared to patients with admission TG > 90.4 mmol/L, those with TG levels between 11.3 and 56.5 mmol/L had significantly lower odds of more severe disease, while no significant differences were found for TG levels between 56.5 and 90.4 mmol/L. Furthermore, neither admission triglyceride levels nor the degree of triglyceride reduction within 72 h was significantly correlated with pancreatic necrosis.

The study involved specific considerations regarding the enrollment timeframe, triglyceride target, and observation window. Unlike recently reported studies [21,22], we enrolled patients within 24 h of symptom onset. Reduced food intake due to abdominal pain, along with fasting status following the diagnosis of acute pancreatitis, can lead to significant changes in triglyceride levels. During fasting, lipoprotein lipase activity in oxidative metabolic tissues increases, allowing triglycerides to be transported to muscles for energy production, thereby resulting in a marked decrease in serum triglyceride levels [23]. Some patients may have received fluid resuscitation before the diagnosis of acute pancreatitis or triglyceride measurement, which can cause a hemodilution effect and affect the accuracy of triglyceride test results. For patients enrolled more than 24 h after symptom onset, the triglyceride levels measured at admission may have already been underestimated.

When serum triglyceride levels exceed the threshold of 5.65 mmol/L, the risk of developing acute pancreatitis increases significantly [17]. Maintaining triglyceride levels below this threshold can effectively prevent the recurrence of HTG-AP [18]. Corresponding endocrine guidelines on the management of hypertriglyceridemia indicate that pharmacological treatment may not be necessary when triglyceride levels are below this threshold [19]. Therefore, in the acute phase of treatment, reducing triglyceride levels below this threshold is considered an indicator of having exited the danger zone. When serum triglyceride levels fall below 5.65 mmol/L, if enteral nutrition is not feasible, parenteral nutrition with minimal lipid content may be cautiously introduced [24]. This recommendation further supports the use of 5.65 mmol/L as a critical threshold in clinical management.

The 72 h period following the onset of acute pancreatitis represents a very early stage and is also a critical period for disease evolution, during which clinical status is closely associated with the final prognosis [25]. During this phase, local pancreatic inflammation may progress to systemic inflammatory response syndrome or even multiple organ dysfunction syndrome [26]. Existing studies [21,22] have also commonly adopted the 72 h window to evaluate the efficacy of early lipid-lowering therapy.

Significant differences in alanine aminotransferase and total bilirubin levels between the two groups are observed in Table 1. Alanine aminotransferase and total bilirubin are common indicators reflecting liver function and the biliary system. In patients with HTG-AP, mild abnormalities in these two indicators might be related to hepatic fatty infiltration associated with hypertriglyceridemia itself, or to reactive changes in the biliary system secondary to inflammation of the adjacent pancreas, rather than indicating an independent biliary etiology. The inclusion criteria of this study excluded other definite etiologies, such as biliary causes, so these differences are unlikely to represent different disease subtypes.

Patients with recurrent HTG-AP were excluded from this study based on the following three considerations. First, patients with recurrent HTG-AP might be admitted multiple times due to repeated acute episodes during the study period. If all these admissions were included in the analysis, the multiple records from the same patient would not be statistically independent. This would artificially inflate the weight of that patient’s specific clinical characteristics in the study sample. Second, these patients may have already received long-term lipid-lowering therapy prior to the index admission. This could directly affect their baseline triglyceride levels upon admission and subsequent treatment responses. Third, the pancreas of patients with recurrent disease may already have chronic structural changes, and their pathophysiology and clinical trajectory differ from those of first-episode patients.

In our study, no statistically significant differences were found between the triglyceride goal-achieved group and the non-goal-achieved group in terms of pancreatic necrosis incidence, length of hospital stay, or in-hospital mortality. These findings are consistent with those of several recent high-quality studies, further suggesting that the clinical value of rapid lipid-lowering therapy in the acute phase remains uncertain. Syed-Abdul et al. [14] systematically evaluated the efficacy of plasmapheresis, insulin, and heparin in the treatment of HTG-AP, concluding that although these interventions effectively reduce triglyceride levels, they do not generally reduce acute pancreatitis-related complications or accelerate disease recovery. These therapies carry risks of adverse reactions, increase resource consumption, and add to healthcare costs. Based on these findings, the authors questioned their routine use. Our observational data, while not designed to evaluate efficacy, do not provide evidence to support a different conclusion. Nevertheless, given the low overall mortality rate in our study, the lack of statistical significance for this endpoint should be interpreted with caution. Another multicenter prospective cohort study of patients with HTG-AP indicated that rapid lipid-lowering therapy may only confer clinical benefit in specific patient subgroups, while, for the overall HTG-AP population, early triglyceride target achievement is not a prerequisite for improved prognosis [27].

In interpreting these null findings, it is important to note the context provided by Table 3. As shown in Table 3, median 72 h triglyceride levels were similar across all three severity groups (approximately 4.16–4.18 mmol/L), all below the 5.65 mmol/L threshold. This indicates that the majority of patients achieved the goal regardless of disease severity. This may partly explain why no significant differences in clinical outcomes were observed between the two groups. Additionally, it should be noted that, as a real-world retrospective study, the sample size of this study was based on available data rather than an a priori power calculation. The low rate of endpoint events observed in this study may, to some extent, reflect the improved prognosis of HTG-AP under modern intensive care support. Therefore, the negative results should not be interpreted as proof that early TG goal achievement is ineffective. Instead, they suggest that the study lacked sufficient evidence to confirm such a difference, given the current sample size and event rate.

Regarding plasmapheresis, the most aggressive lipid-lowering modality, previous studies have shown that it does not accelerate the recovery process of organ failure but may lead to more intensive care unit admissions [28]. An ongoing prospective randomized controlled trial (PERFORM-R) is expected to provide high-quality evidence [29]. In our study, patients in the non-goal-achieved group had a higher proportion of DFPP use, indicating that even with the use of DFPP, triglyceride levels in patients with extremely high baseline TG levels remain difficult to reduce to target within a short timeframe.

Another important finding of this study is that the highest admission triglyceride levels did not correspond to the most severe disease course, although patients with admission triglyceride levels ≥56.5 mmol/L had more severe disease. He et al. [4] demonstrated a nonlinear relationship between the triglyceride-to-high-density lipoprotein cholesterol ratio (TG/HDL-C) and the risk of SAP, with an inflection point at 40. This suggests that the relationship between lipid parameters and disease severity may involve a threshold effect. Feng et al. pointed out through machine learning that TG is one of the most important factors in predicting the severity of HTG-AP [30]. Another study [1] indicated that, at extremely high triglyceride levels, other factors such as genetic predisposition, metabolic stress, and inflammatory response may play a more critical role in disease progression than the absolute triglyceride concentration. A mechanistic study [31] using two genetically modified hyper-triglyceridemic mouse models demonstrated that at the same triglyceride level, chylomicron-rich triglyceride-rich lipoproteins showed greater cytotoxicity than very low-density lipoprotein-rich triglyceride-rich lipoproteins by releasing more free fatty acids. The authors concluded that the subtype of hypertriglyceridemia, rather than the absolute triglyceride level, is a key determinant of acute pancreatitis severity. Long-term prevention of recurrence, rather than rapid triglyceride reduction in the acute phase, might be the cornerstone of HTG-AP management. These perspectives align with our conclusion that simply pursuing early triglyceride reduction goals may be insufficient to improve patient outcomes.

Additionally, neither admission triglyceride levels nor the degree of triglyceride reduction within 72 h was significantly correlated with pancreatic necrosis. Pancreatic necrosis is one of the most severe complications of HTG-AP, and its pathogenesis involving microcirculatory disturbances, inflammatory cascade activation, and pancreatic parenchymal ischemic necrosis [32]. In a retrospective study conducted by Cao et al. [33], independent predictors of HTG-SAP were identified through multivariate binary logistic regression analysis. In their predictive model, admission triglyceride levels were not identified as an independent predictor. This finding is consistent with our observation that triglyceride levels showed no significant correlation with pancreatic necrosis. It also supports the notion that early rapid triglyceride reduction may not be a core determinant of the critical pathological processes underlying HTG-AP. However, our study focused primarily on pancreatic necrosis, hospital stay, and in-hospital mortality as outcome measures. Other clinically relevant endpoints, such as ICU admission, persistent organ failure, and the need for invasive or endoscopic interventions, were not fully evaluated. Future prospective studies should incorporate these endpoints to provide a more complete picture of clinical outcomes.

## 5. Limitations

This study has several limitations. First, the retrospective design may introduce selection bias and confounding by indication, particularly regarding the use of DFPP, which was more commonly applied in patients with higher baseline triglyceride levels. Second, the sample size of patients with extremely high triglyceride levels (>90.4 mmol/L) was relatively small, limiting the precision of estimates in this subgroup. Third, patients with recurrent HTG-AP were excluded from this study. This limits the generalizability of our conclusions to patients with recurrent HTG-AP. Fourth, categorization of triglyceride levels results in a substantial loss of information. The chosen cut-points were based on the diagnostic threshold and equally spaced intervals. Future studies should adopt more precise methods to explore the relationship between triglyceride levels and disease severity. Fifth, the ordinal logistic regression model assumes that the effect of each predictor is consistent across different severity thresholds. While this assumption is standard for this type of analysis, readers should be aware that it represents a simplification of the underlying relationship. Finally, it should be acknowledged that factors such as fluid balance and infusion rate at the time of blood collection, which may affect triglyceride measurements, were not included in the present analysis.

## 6. Conclusions

In conclusion, admission triglyceride levels ≥56.5 mmol/L might be a risk factor for increased disease severity, yet the highest triglyceride levels do not correspond to the most severe disease course. Early triglyceride target achievement was not associated with improved clinical outcomes in this study. The clinical value of aggressive lipid-lowering therapy remains uncertain and requires further investigation in prospective randomized controlled trials. Future prospective studies should investigate whether more intensive therapy in patients with admission triglycerides ≥56.5 mmol/L improves outcomes.

## Figures and Tables

**Table 1:** Characteristics of patients with hyper-triglyceridemic acute pancreatitis on admission (n%; x ± s; median (IQR)).

	TG Goal-Achieved Group (*n* = 274)	TG Non-Goal-Achieved Group (*n* = 71)	*p* Value
Mean age (years)	39.28 ± 10.46	39.76 ± 9.18	0.722
Gender (male%)	207 (75.55%)	49 (69.01%)	0.262
Body Mass Index (kg/m^2^)	27.28 ± 4.13	26.83 ± 4.08	0.407
Previous history			
Hypertension (%)	77 (28.10%)	24 (33.80%)	0.347
Diabetes mellitus (%)	145 (52.92%)	37 (52.11%)	0.903
Drinking (%)	106 (38.69%)	27 (38.03%)	0.919
Current smoke (%)	124 (45.26%)	29 (40.85%)	0.505
Indicators			
White blood cell (×10^9^/L)	10.67 ± 4.47	11.28 ± 4.58	0.308
C-reactive protein (µg/mL)	196.72 ± 104.53	189.38 ± 99.29	0.595
Procalcitonin (ng/mL)	0.64 (1.41)	0.52 (1.21)	0.729
Alanine aminotransferase (U/L)	22.00 (17.00)	16.00 (16.50)	0.031 *
Total bilirubin (µmol/L)	17.00 (12.00)	13.80 (12.73)	0.002 *
Albumin (g/L)	34.86 ± 5.16	35.10 ± 5.70	0.730
Blood urea nitrogen (mmol/L)	3.90 (2.80)	3.60 (2.75)	0.388
Serum creatinine (µmol/L)	62.00 (22.00)	61.50 (27.00)	0.328
Blood glucose (mmol/L)	10.55 ± 4.21	10.59 ± 5.13	0.956
Lactic acid (mmol/L)	2.03 ± 0.94	2.08 ± 0.96	0.727
Triglyceride (mmol/L)	29.27 (33.43)	23.80 (34.86)	0.744
Modified Marshall score	0.00 (2.00)	0.00 (2.00)	0.488
APACHE II score	4.00 (5.00)	5.00 (5.00)	0.103
TG-lowering interventions			
Fenofibrate (%)	180 (65.69%)	44 (61.97%)	0.558
Heparin/LMWH (%)	175 (63.87%)	49 (69.01%)	0.418
Insulin (%)	209 (76.28%)	57 (80.28%)	0.474
DFPP (%)	2 (0.73%)	5 (7.04%)	<0.001 *

Note: APACHE II score: Acute Physiology and Chronic Health Evaluation II score; LMWH: low-molecular-weight heparin; DFPP: Double-filtration plasmapheresis. * *p* < 0.05 was considered statistically significant.

**Table 2:** Prognosis of patients in the TG goal-achieved group versus the non-goal-achieved group after early TG-lowering interventions (n%).

	TG Goal-Achieved Group (*n* = 274)	TG Non-Goal-Achieved Group (*n* = 71)	*p* Value
Pancreatic necrosis			0.957
None	213 (77.74)	56 (78.87)	
<30%	41 (14.96)	9 (12.68)	
30–50%	13 (4.74)	4 (5.63)	
>50%	7 (2.55)	2 (2.82)	
Length of hospital stay (days)	20.41 ± 18.10	18.14 ± 15.44	0.333
In-hospital mortality	3 (1.09)	1 (1.41)	0.826

Note: TG: triglyceride.

**Table 3:** Influence of triglyceride levels on admission and at 72 h after admission on the severity of hyper-triglyceridemic acute pancreatitis (x ± s; median (IQR)).

	Mild	Moderately Severe	Severe	*p* Value
	(*n* = 125)	(*n* = 154)	(*n* = 66)	
TG on admission	25.52 ± 12.00	44.88 ± 28.48	34.86 ± 21.80	<0.001 *
(mmol/L)	22.36 (17.36)	39.35 (44.83)	28.21 (33.59)	
TG at 72 h after admission	4.40 ± 2.03	4.79 ± 2.89	4.28 ± 1.51	0.914
(mmol/L)	4.18 (1.90)	4.16 (2.83)	4.16 (2.31)	
ΔTG	21.12 ± 12.10	40.09 ± 28.20	30.58 ± 21.84	<0.001 *
(mmol/L)	17.04 (17.56)	35.52 (45.27)	23.42 (33.70)	
Percentage decrease (%)	79.47 ± 12.22	83.80 ± 14.25	82.35 ± 12.40	<0.001 *
	83.05 (15.70)	88.77 (14.61)	86.97 (13.68)	

Note: ΔTG: TG on admission minus TG at 72 h after admission; Percentage decrease: ΔTG/TG on admission × 100%. * *p* < 0.05 was considered statistically significant.

**Table 4:** Ordinal logistic regression analysis of the association between triglyceride parameters and disease severity.

	Adjusted B (95% CI)	*p* Adjusted
Threshold		
MAP	−7.027 (−10.797, −3.257)	<0.001 *
MSAP	−4.327 (−8.041, −0.613)	0.022 *
SAP	1	
Triglyceride on admission (mmol/L)		
11.3 ≤ TG < 22.6 (*n* = 133)	−2.281 (−3.465, −1.098)	<0.001 *
22.6 ≤ TG < 33.9 (*n* = 74)	−2.358 (−3.587, −1.129)	<0.001 *
33.9 ≤ TG < 45.2 (*n* = 37)	−1.875 (−3.165, −0.585)	0.004 *
45.2 ≤ TG < 56.5 (*n* = 41)	−1.471 (−2.737, −0.205)	0.023 *
56.5 ≤ TG < 67.8 (*n* = 24)	−0.313 (−1.676, 1.050)	0.653
67.8 ≤ TG < 79.1 (*n* = 16)	−0.876 (−2.358, 0.606)	0.247
79.1 ≤ TG < 90.4 (*n* = 6)	−0.957 (−3.030, 1.116)	0.366
TG > 90.4 (*n* = 14)	1	

*p* adjusted: assessed by ordinal logistic regression; adjusted for age, gender, body mass index, previous history, laboratory indicators at admission, including white blood cell, C-reactive protein, procalcitonin, alanine aminotransferase, total bilirubin, albumin, blood urea nitrogen, serum creatinine, blood glucose, and lactic acid. * *p* < 0.05 was considered statistically significant. Note: B: regression coefficients.

**Table 5:** Collinearity diagnostics among candidate variables included in the regression model.

Variable	Tolerance	VIF
Age	0.685	1.460
Gender	0.630	1.588
Body mass index	0.682	1.466
Hypertension	0.807	1.240
Diabetes mellitus	0.742	1.347
Drinking	0.687	1.457
Current smoke	0.629	1.589
White blood cell	0.919	1.088
C-reactive protein	0.722	1.384
Procalcitonin	0.528	1.894
Alanine aminotransferase	0.890	1.123
Total bilirubin	0.902	1.109
Albumin	0.759	1.317
Blood urea nitrogen	0.340	2.942
Serum creatinine	0.483	2.072
Blood glucose	0.652	1.534
Lactic acid	0.918	1.090

Note: VIF: variance inflation factor. VIF < 5 indicates no significant multicollinearity.

## Data Availability

Data that support the findings of this study are available from the corresponding author upon reasonable request.

## Supplementary Materials

The following supporting information can be downloaded at https://www.mdpi.com/article/10.3390/jcm15124581/s1, STROBE Statement—checklist of items that should be included in reports of observational studies.

## Abbreviations

HTG-AP	Hyper-triglyceridemic Acute Pancreatitis
TG	Triglyceride
MAP	Mild Acute Pancreatitis
MSAP	Moderately Severe Acute Pancreatitis
SAP	Severe Acute Pancreatitis
SD	Standard deviation
IQR	Interquartile range
DFPP	Double-Filtration Plasmapheresis
